# Knowledge, attitude, and practice toward the prevention of occupational exposure in public health emergencies among nurses in Wuhan

**DOI:** 10.3389/fpubh.2024.1289498

**Published:** 2024-04-05

**Authors:** Yan Liu, Zhili Zhang, Ying Liu

**Affiliations:** Tongren Hospital of Wuhan University (Wuhan Third Hospital), Wuhan, China

**Keywords:** knowledge, attitude, practice, public health, nurse, occupational exposure

## Abstract

**Background and objective:**

Nurses have an essential role in dealing with public health emergencies (PHE). This study explored the knowledge, attitude, and practice (KAP) towards preventing occupational exposure in PHE among nurses in Wuhan.

**Methods:**

This cross-sectional study was conducted in May 2023 to assess the KAP of nurses in Wuhan, China. Questionnaires were created and distributed to evaluate the KAP of nurses and explore the factors associated with KAP. Univariate and multivariate logistic regression analyses were used to assess the association between baseline demographic characteristics and KAP, and structural equation modeling (SEM) was used to explore complex relationships and causal pathways among relevant factors.

**Results:**

A total of 440 valid questionnaires were collected. The mean knowledge, attitude, and practice scores were 11.84 ± 2.37, 39.87 ± 3.10, and 44.05 ± 3.76, respectively. The univariate and multivariate logistic regression analyses revealed that age >50 years old (*p* = 0.039), working experience of 1–3 years (*p* = 0.060) and 4–6 years (*p* = 0.024), participation in PHE training, and scene rescue (*p* < 0.001) were significantly associated with knowledge score. In addition, the attitude of the nurses was significantly related to knowledge scores (*p* = 0.002). Moreover, practice was significantly associated with knowledge scores (*p* = 0.005) and attitude scores (*p* < 0.001). The correlation analysis showed that the practice was significantly associated with knowledge (*r* = 0.336, *p* < 0.001) and attitude (*r* = 0.449, *p* < 0.001).

**Conclusion:**

Nurses exhibited moderate knowledge, relatively positive attitude, and practice, which needed to be improved regarding occupational exposure in PHE. The practice of the nurses could be promoted by paying more attention to the working experience, participation in training and scene rescue in PHE, and their knowledge and attitude.

## Introduction

A public health emergency (PHE) is a situation that poses a significant risk to the health of the general population due to the occurrence of diseases, disasters, or other health threats that require urgent action (1). This can include infectious disease outbreaks, bioterrorism events, natural disasters, and chemical or radiological incidents that have the potential to cause widespread health impacts (1). PHE, such as major infectious disease outbreaks, unidentified group diseases, and other events that seriously affect public health, might occur suddenly, causing severe damage to public health (2). In recent years, various PHE have occurred, including the coronavirus disease 2019 (COVID-2019) and the monkeypox virus infection, both of which posed a severe threat and caused serious harm to people’s health (3, 4) According to available data, as of October 2020, there were 1 million documented deaths with COVID-19 (5). Wuhan city is where the first COVID-19 cases have been reported and the city that was hit the hardest across China.

Nurses, the main force on the frontline of epidemics, are vital in preventing and controlling PHE (6). According to recent reports on the workforce in China, 75.6% of nurses have experience in COVID-19 units, and 49.1% have work experience over 10 months (7). However, nurses face increased occupational exposure to viruses or other pathogens when in close contact with patients with infectious diseases (8), which may result in health repercussions. According to previous studies, the infection rate of nurses with high seniority was lower than that of nurses with low seniority (9), indicating that nurses with high knowledge levels and practice capacities could reduce the risks of occupational exposure. Occupational exposure prevention refers to the strategies, practices, and policies implemented to minimize the risk of workers being exposed to hazardous substances, environments, or processes in their workplace. Research in occupational exposure prevention has included studies on the effectiveness of personal protective equipment, vaccination programs, training and education programs for health workers, and the development of protocols for exposure incident management. Studies have also explored the psychological impact of occupational exposure risks on healthcare workers and the importance of supportive policies and practices to mitigate these risks. Thus, understanding the knowledge level, viewpoints, and behavior toward preventing occupational exposure in PHE among nurses is essential for improving the health and safety of nurses and the general public.

The Knowledge, Attitudes, and Practices (KAP) study is a research approach used to understand and measure the knowledge (K), attitudes (A), and practices (P) of a specific population towards a particular subject through a questionnaire (10). Currently, KAP studies have found extensive application in public health. For instance, a study on the KAP among healthcare staff towards the reporting of adverse drug reactions showed that although there was an appropriate attitude towards the reporting of adverse drug reactions, the actual reporting frequency was low (11). Another study highlighted deficiencies among second-year nursing students in handling large-scale PHE, including gaps in knowledge related to infectious disease, epidemiology, evidence-based practice skills, and problem-solving abilities (12). However, there is a dearth of research on the nurses’ KAP on preventing occupational exposure in PHE. In the context of occupational exposure prevention and public health emergencies, KAP studies play a crucial role in identifying the specific knowledge gaps, attitudes, and practices among healthcare workers that could impact the effectiveness of response and prevention strategies. In this study, knowledge pertains to the nurses’ comprehension and command of pertinent information, guidelines, and professional expertise critical for mitigating occupational exposure during public health emergencies. Attitudes encompass the nurses’ perspectives towards preventing occupational exposure, encapsulating their beliefs, viewpoints, assessments, and emotional responses. Practice entails the concrete actions undertaken by nurses in response to public health crises, embodying the practical application of their acquired knowledge and attitudes within their routine professional activities.

This study aimed to explore the KAP towards preventing occupational exposure in PHE among nurses in Wuhan, which could help protect the nurses from occupational exposure and provide the theoretical foundation for policy formulation and medical resource allocation.

## Methods

### Study design and participants

This cross-sectional study surveyed nurses in the Third Hospital, the Children’s Hospital of Wuhan, the Hubei Provincial Combined Traditional Chinese and Western Medicine Hospital, and the Central Theater Hospital between May 1, 2023 and May 31, 2023. This study followed the Strengthening the Reporting of OBservational studies in Epidemiology (STROBE) reporting guideline. The study was approved by the Ethics Committee of the Third Hospital of Wuhan (approval number: KY2022-046), and informed consent was obtained from all the study participants. The inclusion and exclusion criteria are shown in Table 1. Questionnaires were administered to the study participants by online questionnaire.

**Table 1:** Inclusion and exclusion criteria.

Inclusion criteria	Exclusion criteria
1. Nurses who obtained a certificate of practice and registration	1. Registered nurses who were not on duty during the survey period
2. Working continuously in a tertiary hospital for 6 months or more	2. Nurses who were on duty during the survey period but did not belong to this institution
3. Provided informed consent to voluntarily participate in this study	

### Procedures

**Figure 1:**
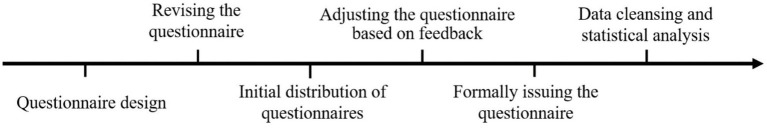
The study flow chart. Flow chart of the questionnaire-based KAP study process.

The procedures used in this study are briefly presented in the flow chart (Figure 1). The questionnaire was designed based on the previously published literature (13, 14) and the Emergency Regulations for PUBLIC HEALTH EMERGENCIES (Revised 2022). A small-scale drop-in was conducted after the questionnaire was designed, reaching Cronbach’s *α* of 0.746 and KMO (Kaiser-Meyer-Olkin) of 0.798.

The final questionnaire was written in Chinese, and it comprised 4 dimensions: (1) demographic information of the participants, which included 7 questions; (2) the knowledge dimensions with a total of 16 questions, where 1–7 were single choice questions with 1 point being assigned for a “clear” answer and 0 points for “unclear” or “unsure” answers; 8–16 were multiple choice questions, where 1 point was assigned for a “correct” answer and 0.5 points for a “partially correct” answer; 0 points for the answer that contained an “incorrect” answer; 0 points for choosing only the “unclear” option; the score range was 0–16 points; (3) the attitude dimension, which consisted of 10 questions evaluated on a five-point Likert scale, with scores ranging from 1–5 points according to the degree of attitude; the score range was from 10–50 points; (4) the practice dimension, which consisted of 10 questions, also evaluated on a five-point Likert scale, with scores ranging from 1–5 according to the degree of action, and a final score range from 10–50 points. Participants with the highest tertile knowledge, attitude, and practice scores were considered to have moderate knowledge, positive attitude, and good practice. The cut-off scores of knowledge, attitude, and practice were 12, 40, and 44, respectively.

In the development of the questionnaire, a preliminary questionnaire was we firstly established by consulting relevant literature. The knowledge section mainly includes: understanding of basic knowledge, occupational protection concepts and knowledge, specific operations and skills, protective equipment, and response measures. The attitude dimension primarily encompasses questions related to cognitive knowledge, practical skills, and emotional-psychological aspects. The practice dimension mainly includes: work environment, exposure to safety and protection, work responsibility and self-management, psychological health, and interpersonal relationships. After designing the questionnaire, it was revised based on feedback from two experts in the field of public health and then distributed on a small scale (38 copies), achieving a reliability of 0.7814.

The questionnaire was developed using a professional online platform, Questionnaire Star, and was distributed via a WeChat-based Questionnaire Star applet, generating a QR code for data collection. Participants logged in and filled out the questionnaire by scanning the QR code sent via WeChat. In order to ensure the quality and completeness of the questionnaire results, the questionnaire could only be submitted once from each IP address, and all items were mandatory. An Excel spreadsheet was exported from the Questionnaire Star platform. The researcher team members checked all the questionnaires for completeness, internal coherence, and rationalization.

The patients in this study were recruited by the head nurse of the nursing committee of each hospital, a total of 6 research assistants participated, and 8 hospitals were surveyed, all of whom were trained in professional nursing, and the questionnaires of the patients were reviewed one by one, and the questionnaires were collected, and the first round of questionnaire cleaning was carried out first, and the waste papers were eliminated. In this study, the Third Hospital of Wuhan City, Wuhan Children’s Hospital, Hubei Provincial Hospital of Integrated Traditional Chinese and Western Medicine, Central Theater Hospital, Hubei Provincial Cancer Hospital, Wuhan Central Hospital, Wuhan Tianyou Hospital, and Zhongxiang People’s Hospital were selected by convenient sampling method, and the questionnaire distribution was 1,000:50 according to the ratio of number, and the final number of questionnaires included was 500. The questionnaire was conducted as an online survey. First, a copy of the questionnaire was edited on the Questionnaire Star platform, including an introduction to the background of the study, the purpose and significance of the study, and the related instructions for filling out the questionnaire. Second, the QR code and link to the questionnaire were sent to the participants through WeChat or QQ. Finally, 440 questionnaires were collected. Participants took approximately 5–10 min to complete the questionnaire, and the questionnaires with >3 items left unfilled were regarded as invalid.

### Statistical analyses

All the statistical analyses were done with SPSS 26.0. Quantitative indicators were presented as mean ± standard deviation (SD). For normally distributed data, *t*-tests or ANOVA were used for intergroup comparisons, while non-normally distributed data were analyzed using the Mann–Whitney or Kruskal–Wallis H-test. Categorical indicators were described using frequencies. Spearman analysis was used to analyze the correlation between knowledge, attitude, and practice scores. Logistic regression was used for univariate and multivariate analysis. A structural equation model (SEM) was performed to test the causal relationships among observed and latent variables using Amos (SPSS plug-in software). The model fit was evaluated by various indices used widely in SEM analysis. *p*-value <0.05 represents a statistically significant difference.

## Results

### Demographic characteristics of the participants

The baseline characteristics are presented in Table 2. A total of 440 valid questionnaires were collected. The mean knowledge score, attitude score, and practice score (mean ± SD) were 11.84 ± 2.37, 39.87 ± 3.10, and 44.05 ± 3.76, respectively (Table 2), suggesting that the participants had moderate knowledge, positive attitude, and good practice. The number of people with KAP scores higher than the mean score was 232 (52.73%), 195 (44.32%), and 215 (48.86%), respectively. The number of people with KAP scores lower than the mean KAP score was 205 (46.59%), 245 (55.68%), and 225 (51.14%), respectively. The highest KAP scores were 16, 50, and 50, respectively (Table 2); the lowest KAP scores were 0, 30, and 29 (Table 2). The respondents were mostly females (96.82%), 31–40 years old (49.32%), with a bachelor’s degree and above (83.86%), with a junior professional title (49.77%), working in a general hospital (88.41%), with working experience of >10 years (49.77%), and have participated in training or scene rescue of PHE (72.95%).

**Table 2:** Baseline data.

Variables	*N* (%)		
	*K*	*A*	*P*
Mean Total Score	11.84 ± 2.37	39.87 ± 3.10	44.05 ± 3.76
Highest Score	16	50	50
Lowest Score	0	30	29
Number of people with scores higher than the mean score	232 (52.73)	195 (44.32)	215 (48.86)
Number of people with scores lower than the mean score	205 (46.59)	245 (55.68)	225 (51.14)
Gender
Male	14 (3.18)		
Female	426 (96.82)		
Age
<30 years old	150 (34.09)		
31–40 years old	217 (49.32)		
41–50 years old	51 (11.59)		
>50 years old	22 (5.00)		
Education level
Junior college and below	71 (16.14)		
Bachelor’s degree and above	369 (83.86)		
Professional title
None	34 (7.73)		
Junior	219 (49.77)		
Intermediate and above	187 (42.50)		
Type of medical institution
General Hospital	389 (88.41)		
Specialty Hospital	40 (9.09)		
Other	11 (2.5)		
Years of working experience
<1 year	33 (7.5)		
1–3 years	32 (7.27)		
4–6 years	50 (11.36)		
7–10 years	106 (24.09)		
>10 years	219 (49.77)		
Have you ever participated in public health emergencies training or scene rescue
Yes	321 (72.95)		
No	119 (27.05)		

### The knowledge of the nurses

The knowledge of the nurses is presented in Table 3. Most participants showed “clear” concern about the concept of PHE (77.73%), the classification of PHE and the time limit for reporting (60.45%), the concept of occupational protection (92.73%), the concept of standard precautions (88.41%), the classification of infectious diseases (79.77%), the emergency response plan and procedures after occupational exposure (90.68%), and the difference between biological, chemical, and physical occupational hazards (61.59%). Regarding the multiple choice questions, most participants had one wrong answer for the factors of biological occupational hazards (wrong answer: c, 53.18%) (Table 3).

**Table 3:** Distribution of responses across dimensions.

3–1 Knowledge	*N* (%)				
	*a*	*b*	*c*	*d*	*e*
1. Are you clear about the concept of public health emergencies?	342 (77.73)	37 (8.41)	61 (13.86)	–	–
2. Are you clear about the classification of public health emergencies and the time limit for reporting?	266 (60.45)	94 (21.36)	80 (18.18)	–	–
3. Are you clear about the concept of occupational protection?	408 (92.73)	12 (2.73)	20 (4.55)	–	–
4. Are you clear about the concept of standard precautions?	389 (88.41)	22 (5)	29 (6.59)	–	–
5. Are you clear about the classification of infectious diseases?	351 (79.77)	26 (5.91)	63 (14.32)	–	–
6. Do you know the emergency response plan and procedures after occupational exposure?	399 (90.68)	16 (3.64)	25 (5.68)	–	–
7. Do you know the difference between biological, chemical and physical occupational hazards?	271 (61.59)	65 (14.77)	104 (23.64)	–	–
8. Which of the following are the factors of biological occupational hazards?	421 (95.68)	417 (94.77)	234 (53.18)	362 (82.27)	9 (2.05)
9. Which of the following are the exposure modes for biological occupational hazards?	402 (91.36)	415 (94.32)	373 (84.77)	374 (85.00)	9 (2.05)
10. Which of the following are the factors of chemical occupational hazards?	416 (94.55)	421 (95.68)	423 (96.14)	414 (94.09)	5 (1.14)
11 Which of the following measures are correct when handling waste that may be infected with germs?	436 (99.09)	120 (27.27)	435 (98.86)	95 (21.59)	1 (0.23)
12. Which of the following are the factors of physical occupational hazards?	171 (38.86)	412 (93.64)	409 (92.95)	340 (77.27)	10 (2.27)
13. What types of masks can be used to against respiratory transmitted diseases during public health emergencies?	368 (83.64)	434 (98.64)	39 (8.86)	87 (19.77)	3 (0.68)
14. By which of the pathways to reduce the risk of occupational exposure are referred to as prevention of occupational exposure?	421 (95.68)	433 (98.41)	412 (93.64)	425 (96.59)	2 (0.45)
15. When caring for a patient with a suspected or confirmed infection, what circumstances require wearing gloves?	421 (95.68)	422 (95.91)	391 (88.86)	278 (63.18)	2 (0.45)
16. Which action is correct when putting on or taking off personal protective equipment (PPE)?	83 (18.86)	174 (39.55)	357 (81.14)	348 (79.09)	5 (1.14)

3–2 Attitude	*a*	*b*	*c*	*d*	*e*
1. Do you think it is important to have a classification of public health emergencies and a time limit for reporting them?	388 (88.18)	47 (10.68)	5 (1.14)	0 (0.00)	0 (0.00)
2. Do you think it is important to know the patient’s medical history beforehand in order to introduce occupational hazards?	363 (82.5)	71 (16.14)	6 (1.36)	0 (0.00)	0 (0.00)
3. Do you think you should be familiar with the classification and types of infectious diseases?	383 (87.05)	50 (11.36)	6 (1.36)	1 (0.23)	0 (0.00)
4. Do you think correctly putting on and taking off protective clothing and isolation gowns is important to reduce occupational hazards?	403 (91.59)	35 (7.95)	2 (0.45)	0 (0.00)	0 (0.00)
5. Do you think it is important for healthcare staff to be vaccinated against Hepatitis B, Influenza and COVID-19 for their own protection?	412 (93.64)	26 (5.91)	2 (0.45)	0 (0.00)	0 (0.00)
6. How important do you think prevention of occupational exposure is in protecting the health of nurses and patients?	413 (93.86)	25 (5.68)	0 (0.00)	1 (0.23)	1 (0.23)
7. Do you think participating in preventing occupational exposure in public health emergencies creates an additional workload for you?	132 (30)	190 (43.18)	36 (8.18)	60 (13.64)	22 (5)
8. Do you feel that your work pressure increases due to occupational exposure in public health emergencies?	48 (10.91)	91 (20.68)	214 (48.64)	40 (9.09)	47 (10.68)
9. Do you feel nervous or scared about occupational exposure in public health emergencies?	112 (25.45)	44 (10)	199 (45.23)	66 (15)	19 (4.32)
10. Do you worry about you or your co-workers contracting a disease?	113 (25.68)	305 (69.32)	5 (1.14)	13 (2.95)	4 (0.91)

3–3 Practice	*a*	*b*	*c*	*d*	*e*
1. Is there a defined rest period and rest area at your workplace?	390 (88.64)	26 (5.91)	19 (4.32)	5 (1.14)	–
2. Do you follow the correct precautions during breaks (e.g., wearing masks correctly, washing your hands regularly, etc.)?	400 (90.91)	27 (6.14)	4 (0.91)	9 (2.05)	0 (0.00)
3. Do you follow up regularly if you have been pricked with a needle from a person with an infectious disease?	363 (82.5)	51 (11.59)	16 (3.64)	10 (2.27)	0 (0.00)
4. Do you pay attention to the properties of various chemical disinfectants and medicines when you come into contact with them?	226 (51.36)	146 (33.18)	44 (10)	22 (5)	2 (0.45)
5. Do you wear gloves and a mask when in contact with chemical disinfectants?	332 (75.45)	93 (21.14)	6 (1.36)	8 (1.82)	1 (0.23)
6. Do you check and distinguish the type of mask before wearing it, e.g., protective mask, or surgical mask?	336 (76.36)	89 (20.23)	12 (2.73)	2 (0.45)	1 (0.23)
7. Would you ignore the operation standard due to your busy work schedule?	54 (12.27)	135 (30.68)	13 (2.95)	113 (25.68)	125 (28.41)
8. Would you take the initiative to learn about public health emergencies?	217 (49.32)	176 (40)	26 (5.91)	19 (4.32)	2 (0.45)
9. When you are frustrated at work, would you talk to others to relieve your bad mood?	120 (27.27)	192 (43.64)	98 (22.27)	23 (5.23)	7 (1.59)
10. When in conflict with others, would you manage your emotions and attitude (speaking loudly, pointing fingers, etc.) to avoid provoking any aggressive behavior from the other person?	196 (44.55)	233 (52.95)	4 (0.91)	7 (1.59)	0 (0.00)

### The attitude of the nurses

The findings on the attitude of participants are provided in Table 3. Most respondents showed strong agreement on the classification of PHE and timely reporting (88.18%), knowing the patient’s medical history beforehand (82.5%), being familiar with the classification and types of infectious diseases (87.05%), the importance to correctly put on and take off protective clothing and isolation gowns (91.59%), the importance of vaccination (93.64%), and the importance of the occupational exposure prevention (93.86%). Less than 1/3 of the participants strongly agreed with the following points: the additional workload brought by participating in the prevention of occupational exposure in PHE (30%), the work pressure increases as a result of occupational exposure in PHE (10.91%), being nervous or scared about occupational exposure in PHE (25.25%), worrying about them or their co-workers contracting a disease (25.68%).

### The practice of the nurses

Most of the participants reported that the following behaviors were always performed or observed (Table 3): a defined rest period and rest area at the workplace (88.64%), following the correct precautions during breaks (90.91%), being followed up regularly if pricked with a needle from a person with an infectious disease (82.5%), paying attention to the properties of various chemical disinfectants and medicines (51.36%), wearing gloves and a mask when in contact with chemical disinfectants (75.45%), checking the mask before wearing it (76.36%), taking the initiative to learn about PHE (49.32%), and managing emotions and attitude to avoid provoking any aggressive behavior (44.55%). Less than 30% of the respondents reported consistently ignoring the operation standard due to their busy work schedule (12.27%) and talking to others to relieve bad mood (27.27%).

### Correlation analysis

The correlation analysis revealed that attitude was significantly associated with the knowledge (*r* = 0.266, *p* < 0.001), and the practice was significantly correlated with the knowledge (*r* = 0.336, *p* < 0.001) and the attitude (*r* = 0.449, *p* < 0.001) (Table 4).

**Table 4:** Correlation analysis.

	Knowledge dimension	Attitude	Behavior
Knowledge dimension	1		
Attitude	0.266 (*p* < 0.001)	1	
Practice	0.336 (*p* < 0.001)	0.449 (*p* < 0.001)	1

### The univariate and multivariate analysis

To determine the factors associated with the KAP of nurses, the univariate and multivariate analyses were carried out; the findings are presented in Table 5. The univariate analysis showed that the knowledge was significantly associated with several factors, including 41–50 years old (*p* < 0.001), >50 years old (*p* = 0.001), with bachelor degree and above (*p* = 0.028), having junior professional title (*p* = 0.002), having intermediate and above professional title (*p* < 0.001), or 1–3 years (*p* = 0.004) or 4–6 years (*p* < 0.001) or 7–10 years (*p* = 0.006) or >10 years (*p* < 0.001) of working experience of, and no participation in PHE training or scene rescue (*p* < 0.001). The results of univariate analysis revealed that participants’ attitudes were remarkably related to the knowledge scores (*p* = 0.002). Moreover, the practice of the respondents was significantly related to the knowledge scores (*p* < 0.001), the attitude scores (*p* < 0.001), and no participation in PHE training or scene rescue (*p* = 0.022) (Table 5).

**Table 5:** Univariate and Multivariate analysis.

Knowledge dimension	Univariate		Multivariate	
	OR (95%CI)	*p*	OR (95%CI)	*p*
Gender
Male	REF			
Female	1.639 (0.540–4.970)	0.383		
Age
<30 years old	REF		REF	
31–40 years old	1.344 (0.879–2.054)	0.173	0.96 (0.45–2.046)	0.916
41–50 years old	3.916 (1.971–7.781)	<0.001	2.338 (0.811–6.744)	0.116
>50 years old	5.547 (1.941–15.856)	0.001	4.378 (1.075–17.835)	0.039
Education level
Junior college and below	REF		REF	
Bachelor’s degree and above	1.810 (1.068–3.070)	0.028	1.315 (0.662–2.613)	0.434
Professional title
None	REF		REF	
Junior	4.874 (1.819–13.06)	0.002	1.673 (0.528–5.298)	0.382
Intermediate and above	7.112 (2.638–19.175)	<0.001	1.766 (0.522–5.969)	0.360
Type of medical institution
General Hospital	REF			
Specialty Hospital	1.571 (0.814–3.033)	0.178		
Other	0.968 (0.290–3.224)	0.957		
Years of working experience
<1 year	REF		REF	
1–3 years	7.778 (1.962–30.826)	0.004	4.172 (0.940–18.512)	0.060
4–6 years	11.739 (3.165–43.536)	<0.001	5.218 (1.240–21.958)	0.024
7–10 years	5.821 (1.666–20.332)	0.006	2.388 (0.555–10.283)	0.243
>10 years	13.298 (3.939–44.89)	<0.001	3.563 (0.741–17.132)	0.113
Have you ever participated in public health emergency training or scene rescue?
Yes	REF		REF	
No	0.214 (0.131–0.348)	<0.001	0.261 (0.155–0.44)	<0.001

Attitude dimension	Univariate		Multivariate	
	OR (95%CI)	*p*	OR (95%CI)	*p*
Knowledge scores	1.139 (1.049–1.237)	0.002	1.139 (1.049–1.237)	0.002
Gender
Male	REF			
Female	0.69 (0.227–2.093)	0.512		
Age
<30 years old	REF			
31–40 years old	0.794 (0.522–1.209)	0.283		
41–50 years old	1.409 (0.723–2.745)	0.313		
>50 years old	0.403 (0.159–1.018)	0.055		
Education level
Junior college and below	REF			
Bachelor’s degree and above	0.788 (0.469–1.322)	0.367		
Professional title
None	REF			
Junior	0.957 (0.462–1.981)	0.906		
Intermediate and above	1.033 (0.495–2.157)	0.931		
Type of medical institution
General Hospital	REF			
Specialty Hospital	0.969 (0.504–1.864)	0.924		
Other	0.951 (0.285–3.169)	0.935		
Years of working experience
<1 year	REF			
1–3 years	1.407 (0.515–3.84)	0.505		
4–6 years	0.938 (0.386–2.279)	0.887		
7–10 years	0.825 (0.375–1.816)	0.633		
>10 years	0.91 (0.434–1.907)	0.802		
Have you ever participated in public health emergency training or scene rescue?
Yes	REF			
No	0.9 (0.59–1.373)	0.625		

Practice dimension	Univariate		Multivariate	
	OR (95%CI)	*p*	OR (95%CI)	*p*
Knowledge scores	1.272 (1.162–1.392)	<0.001	1.159 (1.045–1.285)	0.005
Attitude scores	1.382 (1.273–1.5)	<0.001	1.355 (1.245–1.475)	<0.001
Gender
Male	REF			
Female	0.779 (0.266–2.283)	0.648		
Age
< 30 years old	REF			
31–40 years old	1.03 (0.679–1.562)	0.889		
41–50 years old	1.067 (0.564–2.015)	0.843		
>50 years old	0.542 (0.215–1.367)	0.194		
Education level
Junior college and below	REF			
Bachelor’s degree and above	0.728 (0.436–1.216)	0.225		
Professional title
None	REF			
Junior	1.126 (0.547–2.32)	0.747		
Intermediate and above	0.968 (0.466–2.011)	0.931		
Type of medical institution
General Hospital	REF			
Specialty Hospital	1.088 (0.567–2.088)	0.799		
Other	2.626 (0.686–10.045)	0.158		
Years of working experience
<1 year	REF			
1–3 years	1.771 (0.659–4.761)	0.257		
4–6 years	1.467 (0.606–3.552)	0.395		
7–10 years	0.913 (0.418–1.997)	0.820		
>10 years	1.092 (0.525–2.271)	0.814		
Have you ever participated in public health emergency training or scene rescue?
Yes	REF			
No	1.293(−)	0.022	0.639 (0.383–1.065)	0.085

The factors significantly associated with KAP in the univariate analysis were included in the multivariate analysis. The findings of multivariate analysis showed that the knowledge was significantly associated with several factors including age >50 years old (OR = 4.378; 95%CI, 1.075–17.835; *p* = 0.039), working experience of 4–6 years (OR = 5.218; 95%CI, 1.240–21.958; *p* = 0.024), and no participation in PHE training or scene rescue (OR = 0.261; 95%CI, 0.155–0.44; *p* < 0.001). In addition, the results of the multivariate analysis revealed that attitude was significantly associated with knowledge scores (OR = 1.139; 95%CI, 1.049–1.237; *p* = 0.002). The findings of multivariate analysis showed that the practice was significantly associated with the knowledge scores (OR = 1.159; 95%CI, 1.045–1.285; *p* = 0.005) and the attitude scores (OR = 1.355; 95%CI, 1.245–1.475; *p* < 0.001) (Table 5).

### Path analysis

**Figure 2:**
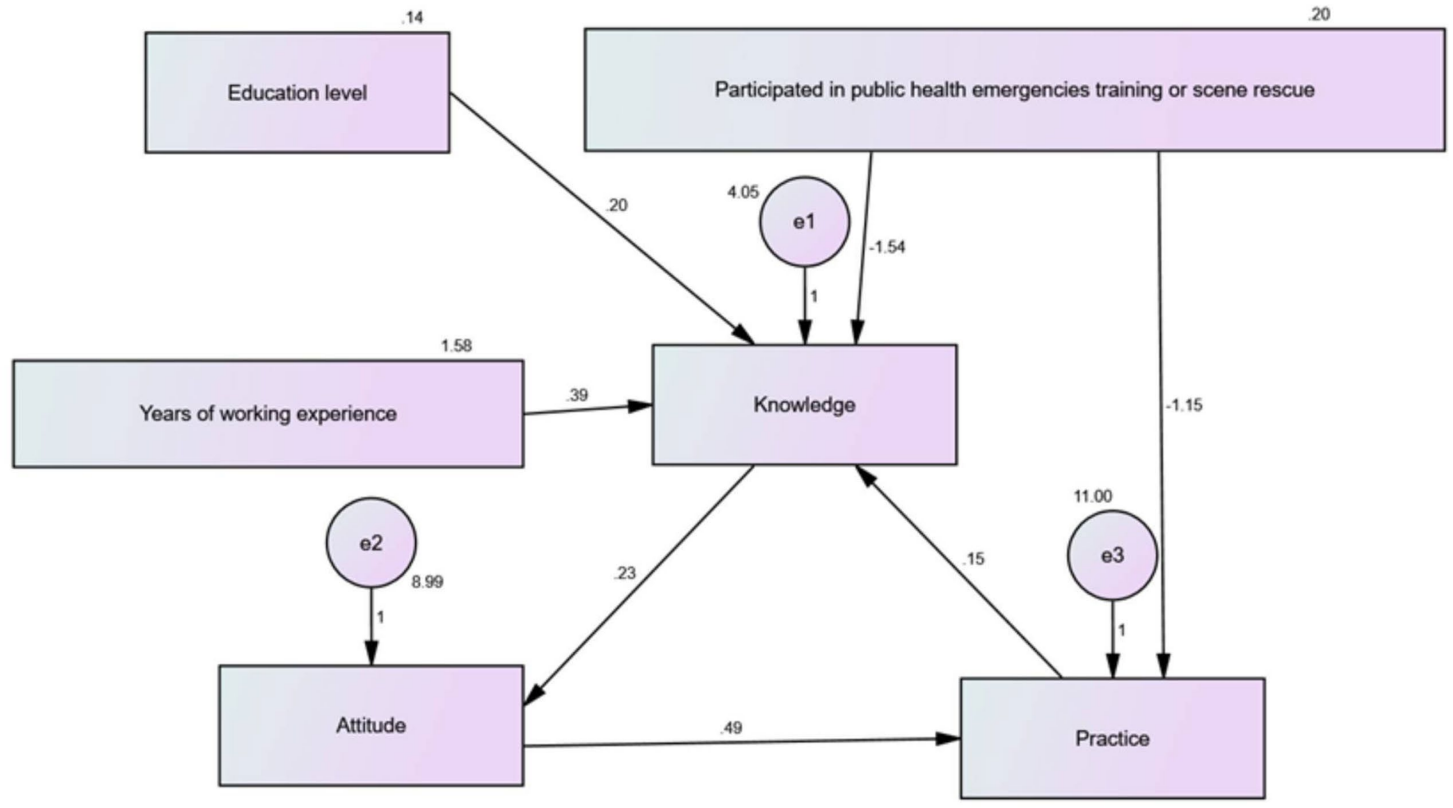
Path diagram of the model. Structural equation model of factors influencing knowledge, attitude, and practice in nursing.

The path analysis was performed, and the path diagram and the effects among the variables are presented in Figure 2 and Table 6, respectively. The results showed that the education level did not significantly exhibit a direct or indirect effect on knowledge, attitude, or practice (*p* > 0.05). Participating in PHE training or scene rescue had direct (path coefficient = −1.544; 95%CI: −1.979, 0.981; *p* = 0.018) and indirect (path coefficient = −0.203; 95%CI: −0.379, 0.097; *p* = 0.004) associations with knowledge and indirect association with attitude (path coefficient = −0.399; 95%CI: −0.716, 0.160; *p* = 0.007). In addition, participating in public health emergency training or scene rescue also had direct (path coefficient = −1.153; 95%CI: −2.016, 0.375; *p* = 0.006) and indirect (path coefficient = −0.194; 95%CI: −0.447, 0.095; *p* = 0.003) associations with practice. Years of working experience had direct (path coefficient = 0.386; 95%CI: 0.223, 0.596; *p* = 0.009) and indirect (path coefficient = 0.007; 95%CI: 0.004, 0.016; *p* = 0.002) effect on knowledge. Besides, years of working experience also indirectly affected attitude (*p* = 0.006) and practice (*p* = 0.003). The attitude showed both direct effect (path coefficient = 0.487; 95%CI: 0.387, 0.605; *p* = 0.008) and indirect effect (path coefficient = 0.008; 95%CI: 0.004, 0.016; *p* = 0.002) on practice, and indirect effect on knowledge (path coefficient = 0.075; 95%CI: 0.045, 0.105; *p* = 0.006). Moreover, the knowledge also had a direct effect (path coefficient = 0.228; 95%CI: 0.133, 0.370; *p* = 0.008) and indirect effect (path coefficient = 0.004; 95%CI: 0.001, 0.010; *p* = 0.004) on attitude, as well as indirect effect on practice (path coefficient = 0.113; 95%CI: 0.055, 0.197; *p* = 0.007).

**Table 6:** The direct and indirect effects among the variables.

			Direct Effects		Indirect Effects	
			Β (95%CI)	*p*	Β (95%CI)	*p*
Knowledge	<−--	Education level	0.198 (−0.300–0.794)	0.438	0.003 (−0.005–0.017)	0.368
Attitude	<−--	Education level	–	–	0.046 (−0.094–0.194)	0.438
Practice	<−--	Education level	–	–	0.022 (−0.043–0.097)	0.395
Knowledge	<−--	Participated in public health emergency training or scene rescue	−1.544 (−1.979–0.981)	0.018	−0.203 (−0.379–0.097)	0.004
Attitude	<−--	Participated in public health emergency training or scene rescue	–	–	−0.399 (−0.716–0.160)	0.007
Practice	<−--	Participated in public health emergency training or scene rescue	−1.153 (−2.016–0.375)	0.006	−0.194 (−0.447–0.095)	0.003
Knowledge	<−--	Years of working experience	0.386 (0.223–0.596)	0.009	0.007 (0.004–0.016)	0.002
Attitude	<−--	Years of working experience	–	–	0.090 (0.036–0.178)	0.006
Practice	<−--	Years of working experience	–	–	0.044 (0.022–0.095)	0.003
Knowledge	<−--	Attitude	–	–	0.075 (0.045–0.105)	0.006
Attitude	<−--	Attitude	–	–	0.017 (0.008–0.028)	0.005
Practice	<−--	Attitude	0.487 (0.387–0.605)	0.008	0.008 (0.004–0.016)	0.002
Knowledge	<−--	Practice	0.151 (0.095–0.199)	0.007	0.003 (0.001–0.005)	0.005
Attitude	<−--	Practice	–	–	0.035 (0.015–0.059)	0.006
Practice	<−--	Practice	–	–	0.017 (0.008–0.028)	0.005
Knowledge	<−--	Knowledge	–	–	0.017 (0.008–0.028)	0.005
Attitude	<−--	Knowledge	0.228 (0.133–0.370)	0.008	0.004 (0.001–0.010)	0.004
Practice	<−--	Knowledge	–	–	0.113 (0.055–0.197)	0.007

### Structural equation modeling

**Figure 3:**
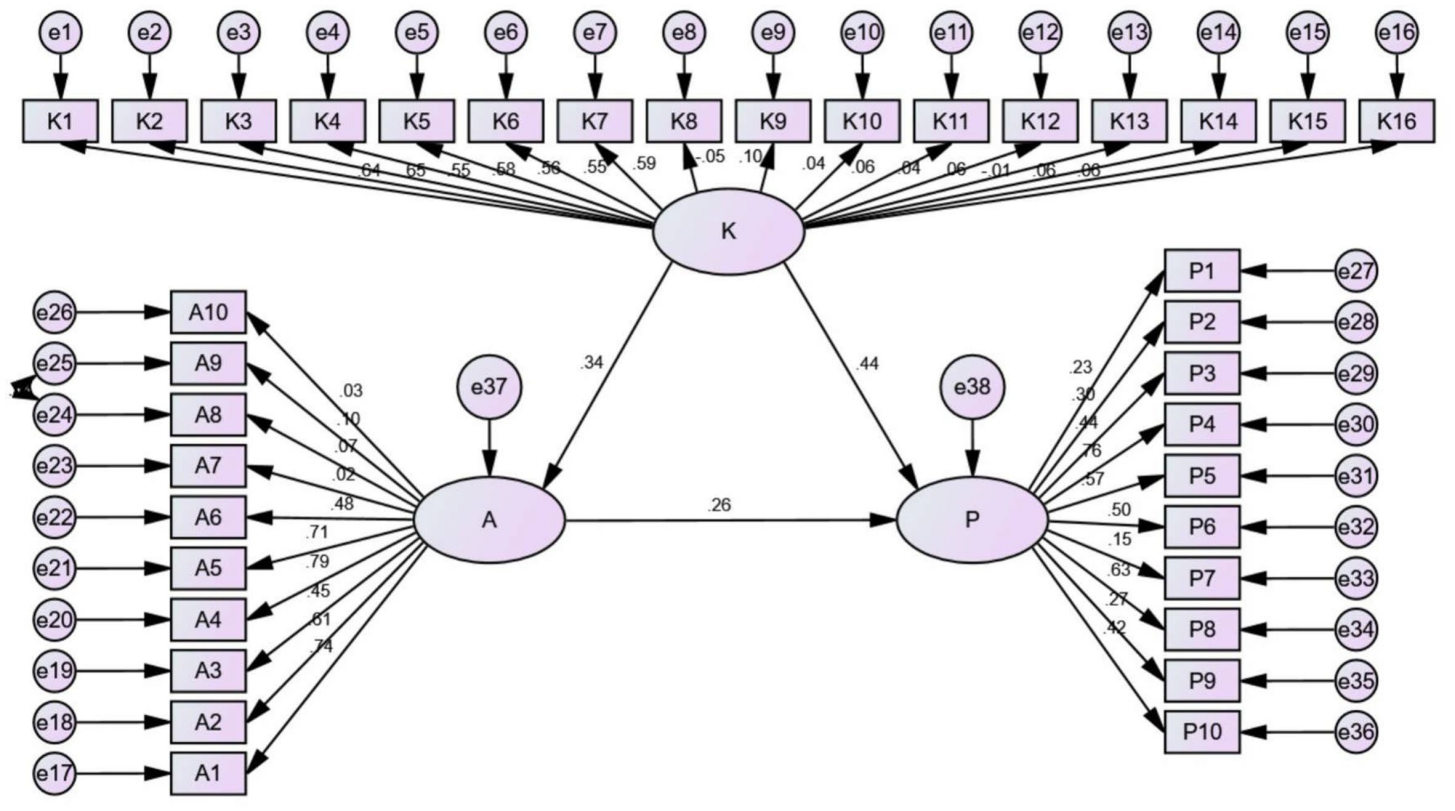
SEM. Detailed SEM diagram of knowledge, attitude, and practice constructs in nursing.

The results of SEM are presented in Figure 3. The results of SEM showed that the path coefficient from knowledge to attitude was 0.34, revealing that nurses’ knowledge could affect their attitude. The path coefficients from attitude to practice and knowledge to practice were 0.44 and 0.26, respectively, suggesting that knowledge and attitude could impact the practice. The value of CMIN/DF in this model was 2.596; in addition, the RMSEA value of the model was 0.06, which is <0.08. Moreover, the regression weight of the model is also presented. The above results demonstrated that the model exhibited excellent function.

## Discussion

Although there have been some studies on the knowledge, attitude, and behavior of medical staff in emergency rescue, there is still a gap in the research on occupational exposure prevention. This study fills this gap and provides new insights and understanding. To the best of our knowledge, this is the first study that evaluated the KAP regarding occupational exposure in PHE among nurses in China. Our findings revealed that the nurses had adequate knowledge, positive attitude, and appropriate practice regarding occupational exposure in PHE. In addition, the nurses’ practice was closely associated with attitude and knowledge. Our findings provide basic theoretical guidance for nurses to prevent occupational exposure or reduce injuries caused by occupational exposure.

The nurses in the present study had adequate knowledge about occupational exposure in PHE. Nevertheless, no more than 2/3 of the participants showed clearness about the classification of PHE, the time limit for reporting, and the difference between biological, chemical, and physical occupational hazards. Those knowledge deficits demonstrate the need for more education about occupational exposure in PHE. The findings of multivariate logistic analysis in this study demonstrated that the knowledge was significantly related to the age >50 years old and working experience of 1–3 years and 4–6 years. These results indicated that older nurses or those with relatively more working experience were more likely to have adequate knowledge about occupational exposure in PHE, which is consistent with previous studies, showing that a nurse with less experience may have insufficient knowledge (15). However, our study also showed that working experience of 7–10 years or >10 years was not significantly associated with knowledge. This might be because nurses with more experience tend to rely more on their experience but not on knowledge, and this point was consistent with the previous study showing that experienced nurses rely more on their intuition (16). The results also showed that the knowledge was significantly associated with no participation in PHE training or scene rescue, indicating that the PHE training or scene rescue might be the main source of improving the knowledge level of nurses, which is in line with the findings of the study conducted by Kim et al. (17). In their study, the nurses who participated in the simulation-based education program showed significantly improved psychological first-aid performance knowledge, competence, and self-efficacy compared to those in the other groups (17). Although a previous study reported that in a county hospital in Jiangxi, China, 21.2% of the nursing staff had a poor level of emergency rescue knowledge, and only 11.9% had a high level of emergency rescue knowledge, this study did not describe the questions investigated or the specific knowledge level of the nursing staff (18). Thus, nurses with more work experience might be better able to deal with occupational exposure, and nurses with less work experience should be provided with more training before they participate in work with a high risk of occupational exposure.

Most of the nurses showed a positive attitude towards occupational exposure in PHE. Some of the respondents held that participating in the prevention program of occupational exposure in PHE creates an additional workload and that occupational exposure in PHE increases work pressure, which might be due to the following: (1) nurses in China are already under substantial working load; (2) the training programs were inefficient. This is consistent with a previous study, which showed that nurses were under great work overload (19) and that the methods that nurse educators taught student nurses theory and clinical skills were inefficient (20). Moreover, some of the nurses in this present study reported feeling nervous or scared about occupational exposure and worried about contracting a disease. This was consistent with a previous study, which showed that nurses in the emergency department were more likely to be exposed to medical dangers (21), demonstrating that nurses facing emergencies might be under pressure due to worry about occupational exposure. According to Hsu et al. (22), anxiety is a feeling of apprehension and uneasiness triggered by uncertainty about a possible threat or worries about unknowns in the future, indicating that the nurses in this study did not have adequate knowledge or could deal with the unknown or complex situations in PHE. Importantly, our findings in SEM revealed that attitude was significantly associated with knowledge. Therefore, improving the knowledge and practice might help alleviate occupational exposure in PHE. Many measures might be beneficial and thus should be implemented. First, it is essential to strengthen the educational programs for nurses and provide higher-level nursing courses and training to improve their professional competence and knowledge. Second, it is advisable to establish a robust training system with standardized training outlines and materials that align with the actual job requirements. Improving training instructors’ qualifications and teaching quality is also important for improving the training outcomes. Third, it is necessary to collaborate with healthcare institutions to provide more internship opportunities and practical training for nurses. This might enable them to learn and accumulate experience in real working environments, thus improving their capabilities and ability to deal with occupational exposure. Finally, it might be beneficial to develop and refine relevant policies and regulations to safeguard the rights and safety of nurses and strengthen supervision and penalties for occupational exposure behaviors, thereby promoting improvements in the working environment. By implementing these comprehensive measures, it is possible to reduce occupational exposure risks among PHE nurses effectively.

The present study showed that the nurses had good practice considering occupational exposure. Although most participants obtained high practice scores, a proportion reported that they usually ignored the operation standard due to busy work schedules, indicating that they might be overloaded at work, which aligns with the results of nurses’ attitudes towards participating in prevention programs of occupational exposure in PHE. In addition, only 27.27% of the participants reported always speaking to others to relieve bad mood, demonstrating that the nurses did not know how to treat pressure or emotion in PHE. A previous study found that 37.3% of the nurses had a low level of emergency rescue practice, and only 6.8% of the participants had a high level of practice (18). However, another study based on literature analysis showed that 33.5% of medical staff had a high level of score in emergency rescue behavior (23). The above studies show that there are large differences in the results of different studies, which may be caused by regional differences, differences in research subjects and differences in research methods. Therefore, appropriate education should be provided to enhance nurses’ capability to deal with occupational exposure. The multivariate analysis findings showed that the practice was significantly associated with the knowledge and attitude scores. In addition, the results of SEM showed that nurses’ knowledge could affect their attitude, and their knowledge and attitude could impact their practice. Therefore, to promote the practice, nurses and leaders of the nursing departments should place greater importance on the factors directly associated with the practice and the factors related to the knowledge and attitude, such as working experience and participation in public health emergency training or scene rescue.

This study has a few limitations. First, this is a cross-sectional study with a relatively small sample size; thus, causality cannot be inferred. Prospective studies with larger sample sizes should be conducted in the future. In addition, data in this study were self-reported, indicating less reliability than other objective research. Besides, this study was conducted in Wuhan, China, limiting the generalizability of the reported findings.

## Conclusion

Our results revealed a close correlation between the behavior of nurses and their knowledge and attitudes, indicating the importance of improving nurses’ education level, training, and experiential learning related to occupational exposure. Although the participants showed adequate knowledge, positive attitudes, and good practice in preventing occupational exposure, more effort is required to enhance their KAP so as to reduce the direct and indirect injuries caused by occupational exposure in PHE. This is the first report on the KAP of nurses in China towards occupational exposure in PHE, and as such, this KAP study holds critical implications for shaping vocational training and elevating nursing care standards, bolstering the emergency preparedness of healthcare institutions, and fortifying the resilience of the health system against crises. Furthermore, it establishes a foundational framework for future research aimed at understanding and improving the interplay between nurses’ knowledge, attitudes, and practices in public health emergencies.

## Data availability statement

The original contributions presented in the study are included in the article/supplementary material, further inquiries can be directed to the corresponding author.

## Ethics statement

The studies involving humans were approved by the study was approved by the Ethics Committee of the Third Hospital of Wuhan (approval number: KY2022-046), and informed consent was obtained from the study participants. The studies were conducted in accordance with the local legislation and institutional requirements. The participants provided their written informed consent to participate in this study.

## Author contributions

YaL: Conceptualization, Data curation, Writing – original draft, Writing – review & editing. ZZ: Data curation, Writing – original draft, Writing – review & editing. YiL: Conceptualization, Writing – original draft, Writing – review & editing.
